# Improvements of weaned pigs barn hygiene to reduce the spread of antimicrobial resistance

**DOI:** 10.3389/fmicb.2024.1393923

**Published:** 2024-05-14

**Authors:** Megarsa Jaleta, Vera Junker, Baban Kolte, Maria Börger, Doreen Werner, Claudia Dolsdorf, Julia Schwenker, Christina Hölzel, Jürgen Zentek, Thomas Amon, Ulrich Nübel, Tina Kabelitz

**Affiliations:** ^1^Leibniz Institute for Agricultural Engineering and Bioeconomy (ATB), Potsdam, Germany; ^2^Dahlem Research School, Freie Universität Berlin, Berlin, Germany; ^3^Leibniz-Institute DSMZ—German Collection of Microorganisms and Cell Cultures, Braunschweig, Germany; ^4^Technical University Braunschweig, Institute of Microbiology, Braunschweig, Germany; ^5^Leibniz Centre for Agricultural Landscape Research (ZALF), Müncheberg, Germany; ^6^Teaching and Research Station for Animal Breeding and Husbandry (LVAT), Ruhlsdorf, Germany; ^7^Faculty of Agricultural and Nutritional Sciences Christian-Albrechts-University of Kiel, Kiel, Germany; ^8^Institute of Animal Nutrition, Free University Berlin, Berlin, Germany; ^9^Institute for Animal Hygiene and Environmental Health (ITU), Free University Berlin, Berlin, Germany; ^10^German Center for Infection Research (DZIF), Partner Site Braunschweig-Hannover, Braunschweig, Germany

**Keywords:** AMR, cultivation, disinfection, Escherichia coli, hygiene, pig, weaner barn

## Abstract

The spread of antimicrobial resistance (AMR) in animal husbandry is usually attributed to the use of antibiotics and poor hygiene and biosecurity. We therefore conducted experimental trials to improve hygiene management in weaned pig houses and assessed the impact on the spread. For each of the two groups examined, the experimental group (EG) and the control group (CG), three replicate batches of piglets from the same pig breeder, kept in pre-cleaned flat decks, were analyzed. In the flat decks of the experimental groups, the hygiene conditions (cleaning, disinfection, dust removal and fly control) were improved, while regular hygiene measures were carried out in the control groups. The occurrence and spread of AMR were determined in *Escherichia coli* (*E. coli*; resistance indicator) using cultivation-dependent (CFU) and -independent (qPCR) methods as well as whole genome sequencing of isolates in samples of various origins, including feces, flies, feed, dust and swabs. Surprisingly, there were no significant differences (*p* > 0.05) in the prevalence of resistant *E. coli* between the flat decks managed with conventional techniques and those managed with improved techniques. Selective cultivation delivered ampicillin- and sulfonamide-resistant *E. coli* proportions of up to 100% and 1.2%, respectively. While 0.5% *E. coli* resistant to cefotaxime and no ciprofloxacin resistance were detected. There was a significant difference (*p* < 0.01) in the abundance of the *bla_TEM-1_* gene in fecal samples between EG and CG groups. The colonization of piglets with resistant pathogens before arrival, the movement of flies in the barn and the treatment of bacterial infections with antibiotics obscured the effects of hygiene improvement. Biocide tolerance tests showed no development of resistance to the farm regular disinfectant. Managing hygiene alone was insufficient for reducing antimicrobial resistances in piglet rearing. We conclude that the complex factors contributing to the presence and distribution of AMR in piglet barns underscore the necessity for a comprehensive management strategy.

## Introduction

Antimicrobial resistance (AMR) is one of the leading causes of morbidity and mortality worldwide. It has a huge negative impact on economic sustainability (FAO, UNEP, WHO, and WOAH, 2020; Walsh et al., 2022). The large worldwide use of antimicrobials for animals (73% of all administered antibiotics) is correlated with increased AMR spread in humans and animals (Boeckel et al., 2017; Ardakani et al., 2023). On one hand, this could be due to the fact that certain antibiotic classes are used in animals and humans [Rahman and Hollis, 2023; European Food Safety Authority (EFSA) and European Centre for Disease Prevention and Control (ECDC), 2024]. On the other hand, it could be because resistant pathogens and/or genes can be transmitted to humans through food chains or direct contact with livestock (Bennani et al., 2020). This alarming trend underscores the need for effective resistance control and prevention strategies to protect human and animal health and sustain the economy.

In order to effectively combat resistance spread, a comprehensive understanding of the emergence and transmission of resistance is crucial (Berendonk et al., 2015). This can be possible through phenotypic and genotypic surveillance and monitoring of resistance patterns in bacterial populations over time (Graham et al., 2019). Both are very useful for understanding the resistance mechanisms and are important for developing strategies against the spread of antibiotic-resistant bacteria and protecting public health (Diallo et al., 2020).

*Escherichia coli* (*E. coli*) is commonly used as an indicator organism in many antimicrobial resistance surveillance programs (Anjum et al., 2021; Safety, European Food, and European Centre for Disease Prevention and Authority Control, 2021). Because *E. coli* is a potential reservoir and transmitter of many different AMR genes. As a commensal bacterium in the intestines of humans and animals, it also facilitates the acquisition and accumulation of resistance genes (Pissetti et al., 2021). Similarly, *E. coli* is ubiquitous and is found in various environments including the gastrointestinal tract, water, soil, and food (Jang et al., 2017). These properties make it a valuable sentinel organism for monitoring AMR trends and transmission in various environments. Hence, by tracking the presence and patterns of AMR in *E. coli*, surveillance programs can provide valuable insight into the overall prevalence and spread of AMR, thereby supporting the development of combating strategies to this global health threat (Suwono et al., 2021).

In pig production, antibiotics are primarily used to treat and combat diseases (Cromwell, 2002; Hallenberg et al., 2020). They are particularly often applied in the early weaning period, as the piglets are very vulnerable for infections (Dewulf et al., 2022). Weaning, which is conventionally done at 3 to 4 weeks of piglet age, is an extremely stressful time marked by dietary and environmental changes, often resulting in decreased feed intake, inadequate weight gain, and potential problems such as diarrhea, morbidity, and mortality. During this period, piglets are very susceptible to infections due to their underdeveloped immune system, compounded by the rapid decline in maternal passive immunity and the early onset of active immunity (Campbell et al., 2013).

The status of AMR resistance varies across pig farms depending on antibiotic usage, production type, hygiene practices, and biosecurity measures (Österberg et al., 2016; Mencía-Ares et al., 2021). There are studies indicating that farmers use large amounts of antibiotics to combat diseases associated with poor biosecurity and unhygienic conditions in farms (Raasch et al., 2018; Albernaz-Gonçalves et al., 2021). Incorrect and excessive use of antibiotics contributes to the widespread emergence and spread of AMR in livestock farms (Aarestrup et al., 2008; Holman and Chénier, 2015; EMA and EFSA, 2017). Although, AMR occurrence can be decreased by reducing antimicrobial use (Friedman and Whitney, 2008; European Centre for Disease Prevention and Control (ECDC) et al., 2017). The spread of AMR can also be counteracted through strict biosecurity and hygiene management (Laanen et al., 2013; Dohmen et al., 2017; Raasch et al., 2018).

In our preliminary work (Behrens et al., 2023), a dramatic increase in the prevalence of resistant bacteria (from <1% of resistant *E. coli* to almost 100%) in fattening piglets was observed within the first 4 weeks after weaning at farm arrival. This was not solely due to selection pressure from the use of antibiotics alone, but also possibly due to the colonization of antimicrobial-resistant microbes from the piglet environment in the barn (Yun et al., 2021; Saladrigas-García et al., 2022; Smith et al., 2023). In addition to the use of antimicrobial medications and management, factors such as barn hygiene can influence the emergence and persistence of drug-resistant intestinal bacteria in pigs (Dewulf et al., 2007). Some reviews also speculate about a connection between biocide and antibiotic resistance in bacteria, particularly through the mechanism of numerous efflux systems that cause co-(cross-)resistance to a number of structurally unrelated antimicrobials such as antibiotics and biocides (Ortega et al., 2013; Jean-Yves, 2018). Similarly, Puangseree et al. (2021) recommend routine monitoring of biocide tolerance because it plays a role in the emergence and spread of AMR when used frequently, acting as a non-antibiotic selection pressure. By implementing effective hygiene measures such as thorough cleaning, disinfection, and biosecurity measures, it is possible to curb the development and reduce the spread of AMR (Yun et al., 2021). In this study, we improved hygiene in the piglet barn through increased hygiene management techniques and compared the impact on the spread of AMR with routine practices. We analyzed biocide resistance of barn AMR *E. coli* to the farm regular disinfectant that had been used in the study farm for many years.

## Materials and methods

### Experimental procedures

The study was conducted at the Teaching and Research Institute for Animal Breeding and Animal Husbandry (LVAT) in Ruhlsdorf, Germany, from April to September, 2022. For more information about the farm operation, barn design and records of the antibiotic usage during the study period (see Supplementary material). In this study, we examined the effects of two hygiene management methods on the spread of antimicrobial resistance in three experimental groups and three control groups, each containing of 20 to 40 piglets, depending on availability from the supplier.

The flat decks of the experimental groups were cleaned using improved techniques before the piglets arrived, while the houses of the control groups were cleaned using the farm routine hygiene management technique. All flat decks had the same area of about 18 m^2^ (length 6 m, width 3 m), but has a variable number of pens (2–4) (Supplementary Figure S1). In the experimental groups (EG): all solid dirt was scraped from the floor, walls, and surfaces, and the dust was removed. The remaining flies in the house were killed by spraying a natural and environmentally friendly insecticide (Flybuster Spray, Steel Agro GmbH, Edewecht, Germany); approx. 40–50 sprays in each use. The flat deck surfaces with feces residuals were soaked with a 2% solution of an alkaline cleaning agent DESINTEC®FL-R1 (AGRAVIS Raiffeisen AG, Münster, Germany) and remained for 20 min. Afterward, the flat deck was cleaned with high-pressure (150 kPa) water. After 30–40 min of roughdrying, the flat deck was disinfected with DESINTEC FL-des Allround (AGRAVIS Raiffeisen AG, Münster, Germany) according to the manufacturer’s instructions. DESINTEC®FL-des Allround is a broad-spectrum disinfectant with two active ingredients: Comp. A is 2-hydroxyphenyl and Comp. B is peracetic acid. The concentrations of the disinfection components used in this experiment were 3% (≈28.9 ppb) for Comp. A and 1.5% (≈14.5 ppb) for Comp. B. After piglet arrival, hygiene monitoring was done once a week by wet dusting, checking fly population, and insecticide spraying, if needed. Once a week, accumulated feces were scraped into the pit below the floor and the flat deck-specific disinfection footbath kept at the flat deck entrance was controlled.

Hygiene management in the flat deck of the control group (CG) was performed according to the farm conventional procedure and is explained in the following: First, dry cleaning and subsequent soaking with water without a detergent was executed. After 20 min, cleaning with water under high pressure was performed. The dried flat deck was disinfected with 2% (≈19.8 ppm) of Sorgene®Xtra (BASF SE Pest Control Solutions GmbH, Ludwigshafen, Germany) according to the manufacturer’s instructions. It is a stabilized mixture of peracetic acid and hydrogen peroxide. The fly population was controlled using sticky baits. Goldin (rotie-pharm GmbH & Co. KG, Osnabrück, Germany) was used during fly peak times, especially in summer.

### Sample collection

To determine the efficiency of cleaning and disinfection procedures, swab samples from cleaned flat deck surfaces were collected before piglet arrival using hydrated sponges (3 M Deutschland GmbH, Neuss, Germany), particularly from the walls, floors, and feeding troughs. After the arrival of the piglets (within 12 to 24 h), a pooled fecal sample, feed sample, and fly sample (three flies caught in the flat deck) were collected to test the presence of AMR *E. coli* in the piglets and their environment upon arrival. Subsequently, samples were collected weekly (week one, week two, and week four after piglet arrival) from both the control and experimental groups. These samples included pooled feces, feed, dust, and three flies for each time point and group. The sampling procedures were as follows: in each flat decks as described in Behrens et al. (2023) approximately 10–15 fresh fecal drops were collected from multiple locations within the piglet pens using a sterile spatula into sterile 120 mL propylene containers. Individual flies were captured alive using sterile polypropylene tubes. The deposited dust was collected with sterile cotton gauze from the surfaces (i.e., the small roof above the piglet lying areas and the window sills) into sterile 125 mL polypropylene containers (AMPri Handelsgesellschaft mbH, Germany). These weekly samples were collected to monitor the emergence and temporal dynamics and spread of resistant *E. coli* throughout the experiment. To ensure the preservation and integrity of the samples, proper cold chain conditions (≈4°C) were maintained during sample transportation to the lab.

### Selective cultivation

For feces and feed samples 10–15 grams and for dust 5–10 grams were placed in a stomacher bag, mixed 1% phosphate-buffered saline (PBS, ≈ 0.09 M; Roth, Karlsruhe, Germany) in a ratio of 1:5, and homogenized for 30 s. Aliquots were serially diluted 10-fold up to six orders of magnitude. Three cold-shocked flies were placed in a sterile 1.5 mL Eppendorf tube and crushed with a disposable polypropylene microtube pestle and first diluted 1:5 with PBS and then serially diluted 10-fold up to six orders of magnitude. 50 μL of each dilution was plated on MacConkey Agar No.3 plates (MC3; Oxoid, München, Germany), with and without antibiotics. The antibiotics selected were based on the class of antibiotics used in the farm. The antibiotic solution were added to MC3 agar medium (≈ 50°C) and mixed well before pouring the plates. Tested antibiotics and concentrations were: ampicillin (10 mg/L), cefotaxime (1 mg/L), ciprofloxacin (0.5 mg/L), and sulphonamide (512 mg/L; all Sigma-Aldrich, Darmstadt, Germany) based EUCAST Clinical Breakpoint Tables v. 12.0, valid since 2022-01-01. The plates were incubated in aerobic conditions overnight (18–20 h) at 44°C. We used this incubation temperature based on the higher temperature tolerance of *E. coli* compared to other *Enterobacteriaceae.* 44°C supported the growth of *E. coli* but restricted the growth of other lactose-fermenting *Enterobacteriaceae* (Irrgang et al., 2019) and facilitated quantification of *E. coli* colonies. Plates with colonies from 30 to 300 colony-forming units (CFUs) were counted for analyses. To confirm the isolate bacterial species, PCR using *E. coli*-specific primers from Torres et al. (2017) was performed. Up to eight isolates per positive antibiotic plate were preserved for further analyses. Mean values and the proportion of resistant to non-resistant *E. coli* were calculated from the CFU counts at each sampling time. We checked quantitative cultivation results for statistically significant differences by nonparametric pairwise testing with the Wilcoxon signed rank test using the R (version 4.2.3) package *ggpubr*.

### Genome sequences and resistance determinants to common antibiotics

A total of 68 resistant *E. coli* strains isolated (based on selective cultivation on selective MC3 No. 3 with antibiotics) from fecal samples and 6 from swab samples were selected for whole-genome sequencing. DNA was extracted using the DNeasy Blood and Tissue Kit (Qiagen, located in Hilden, Germany) and miniaturized Nextera XT protocol was used to prepare the libraries for sequencing (Baym et al., 2015). The DNA was sequenced using a NextSeq 550 instrument with a NextSeq 500/550 mid-output v2.5 kit from (Illumina). After demultiplexing, the obtained sequence data were uploaded to the Enterobase database^1^ (Zhou et al., 2020), where it underwent automatic assembly and quality-checking procedures. The assembled genome was downloaded from Enterobase and the AMR genes determinants were identified using the Resistance Gene Identifier based on the Comprehensive Antibiotic Resistance Database (CARD: https://card.mcmaster.ca/analyze/rgi; Alcock et al., 2023). Genome sequence analyses were performed as described in Behrens et al. (2023).

### Quantification of antimicrobial resistance genes in feces samples (ARGs–qPCR)

DNA was extracted in duplicates from 24 fecal samples (12 from control groups and 12 from experimental groups; 0.2 g for each duplicate), using the modified QIAamp Fast DNA Stool Mini Kit (Knudsen et al., 2016). Based on the frequency in sequenced isolates (data not shown) and the antibiotics used in the farm, four antimicrobial resistance genes (ARGs) have been selected for quantification, *bla_CTX-M-1_*, *bla_TEM-1_*, *sul2,* and *tet(A)*. The *16S rRNA* housekeeping gene (*Com1 R789*) was included for normalization and as a proxy for the total bacterial load in the feces samples. Quantitative PCRs were executed in triplicates and the gene copy numbers for every sample were calculated as the mean of six measurements (2 extraction duplicates × 3 qPCRs triplicates). qPCRs were performed using a LightCycler 480 Instrument II (Roche Diagnostics Deutschland GmbH). Primer sequences were designed for *bla_CTX-M-1_* and *bla_TEM-1_*, while for *16S rRNA*, *sul2*, and *tet(A)* the primer sequences were obtained from literature (Table 1). PCR products of the selected genes were 10-fold serially diluted and used for standard curve measurements. Reactions were performed in total volumes of 18 μL, containing 10 μL of SYBR Green Master I (Light Cycler 480 SYBR Green I Master, Roche), 1 μL of each forward and reverse primer (10 mM), 5 μL of PCR-grade nuclease-free water and 1 μL of DNA template. A PCR-master mix without a template served as a negative control. The qPCR program consisted of a pre-incubation cycle (95°C, 30 s), 40 amplification cycles (95°C, 10; 60°C, 10 s annealing temperature; elongation at 72°C, 10 s, and a final cycle for melting curve acquisition; 95°C, 5 s; 65°C, 60 s; 95°C followed by cooling at 40°C for 10 s).

**Table 1:** Selected primer pairs for quantification of ARGs in fecal samples from piglets.

List no.	Primer name	Sequence (5’-3’)	Amplicon size (bp)	GenBank accession no.	Reference
1	*TEM-1*_F	GGGAACCGGAGCTGAATGAA	188	KP634895	This study
	*TEM-1*_R	CAGTGCTGCAATGATACCGC			
2	*sul2_F*	GATATTCGCGGTTTTCCAGA	141	-	Schmidt et al. (2015)
	*sul2_R*	CGCAATGTGATCCATGATGT			
3	*CTX-M-1*_F	GGTGACTATGGCACCACCAA	109	KP634890	This study
	*CTX-M-1*_R	GACGGCTTTCTGCCTTAGGT			
4	*tet(A)_F*	TTGGCATTCTGCATTCACTC	125	-	Schmidt et al. (2015)
	*tet(A)_R*	GAAGGCAAGCAGGATGTAGC			
5	*Com1 R789_F*	CAGCAGCCGCGGTAATAC	270	-	Bassitta et al. (2022)
	*Com1 R789_R*	ATCCTGTTTGMTMCCCVCRC			

M = Adenine/Cytosine, V = Guanine/Cytosine/Adenine, R = Guanine/Adenine.

### MIC determination of disinfectants

A broth macrodilution test was carried out according to the method of the German Veterinary Medical Association (DVG, 2017) to determine the susceptibility of *E. coli-*isolates from the control and experimental groups. We randomly chose 25 *E. coli* per group (control / experiment, week 0) from samples that did not show growth of *E. coli* on cefotaxime-supplemented agar (1 mg/L). In addition, we examined 13 isolates grown on cefotaxime agar (1 mg/L) and confirmed by PCR. In addition, by random selection, some isolates grown on CTX-containing plates were classified as resistant by the E test. The isolates were subcultured on Columbia blood agar with 5% sheep blood (Thermo Fisher Scientific, Waltham, Massachusetts, USA) and incubated aerobically at 37°C for 24 h. The reference strain *E. coli* DSM 1103 (collection no: ATCC 25922) were also included in the test. The bacterial test suspension was prepared according to CLSI (2015). Colony material was collected with a sterile swab (ROTILABO®; Carl Roth GmbH & Co. KG, Karlsruhe, Germany) and was adjusted to a turbidity equal to 0.5 McFarland standard (Carl Roth GmbH & Co. KG, Karlsruhe, Germany) in sodium chloride, corresponding to approximately 1.5 × 10^8^ CFU/mL. A 1:10 dilution in tryptone sodium chloride was prepared and 100 μL of a final bacterial concentration of 1.5 × 10^7^ cfu/mL was added to each test tube. The two disinfectants (Sorgene®Xtra and DESINTEC FL-des Allround) were diluted (undiluted, 1:10, 1:100, 1:1,000, and 1:10,000) with water of standardized hardness as in Schwenker et al. (2022) described. 500 μL of the disinfectant solution was added to 4.5 mL of Trypton soya broth to make a 1:10 dilution in a macrodilution tube. Test tubes were incubated at 37°C for 72 h and vortexed daily. The MIC value, defined as the lowest disinfectant concentration without visible bacterial growth (clear tube), was assessed after 72 h of incubation. To check the test tubes for contamination and in the case of the MIC—the absence of growth, 100 μL of the test tubes were streaked onto blood agar and incubated again at 37°C for 24 h. The result were interpreted based on the German Veterinary Society (DVG, 2017) guideline.

## Results

### Selective cultivation of AMR *Escherichia coli*

In this study, a total of 88 samples (i.e., feces, flies, feed, swabs, and dust) were plated on MacConkey agar (MC3) with and without antibiotics (Table 2). *Escherichia coli* colonies grown on plates containing antibiotics (subsequently confirmed by E-test and whole genome isolate sequencing) were detected most frequently for ampicillin and sulfonamide. Only a few samples contained *E. coli* resistant to cefotaxime, while no ciprofloxacin resistance was detected in all samples tested. AMR *E. coli* grew from most fly and feed samples on ampicillin plates, but only about 12% of the fly and feed samples contained sulfonamide-resistant *E. coli*. Likewise, 12.5% of the 16 dust samples showed ampicillin-resistant *E. coli*, while none were resistant to cefotaxime and ciprofloxacin in the fly, feed, and dust samples. No sulfonamide-resistant colonies were detected from dust samples (Table 2).

**Table 2:** Quantity and percentage of samples with *Escherichia coli* colony growth on indicated antibiotic containing plates (% CFU).

Sample	Total no.	Ampicillin-plates		Sulfonamide-plates		Cefotaxime-plates		Ciprofloxacin-plates	
		EG	CG	EG	CG	EG	CG		CG
Feces	24	12(100%)	12(100%)	9(75%)	11(92%)	5(42%)	0	0	0
Flies	24	11(92%)	7(58%)	2(17%)	1(8%)	0	0	0	0
Feed	24	7(58%)	8(67%)	2(17%)	1(8%)	0	0	0	0
Dust	16	1(8%)	3(25%)	0	0	0	0	0	0
Total	88	31(65%)	30(63%)	13(27%)	13(27%)	5(5.7%)	0	0	0

EG, Experimental group with improved hygiene; CG, Control group with conventional hygiene.

**Figure 1:**
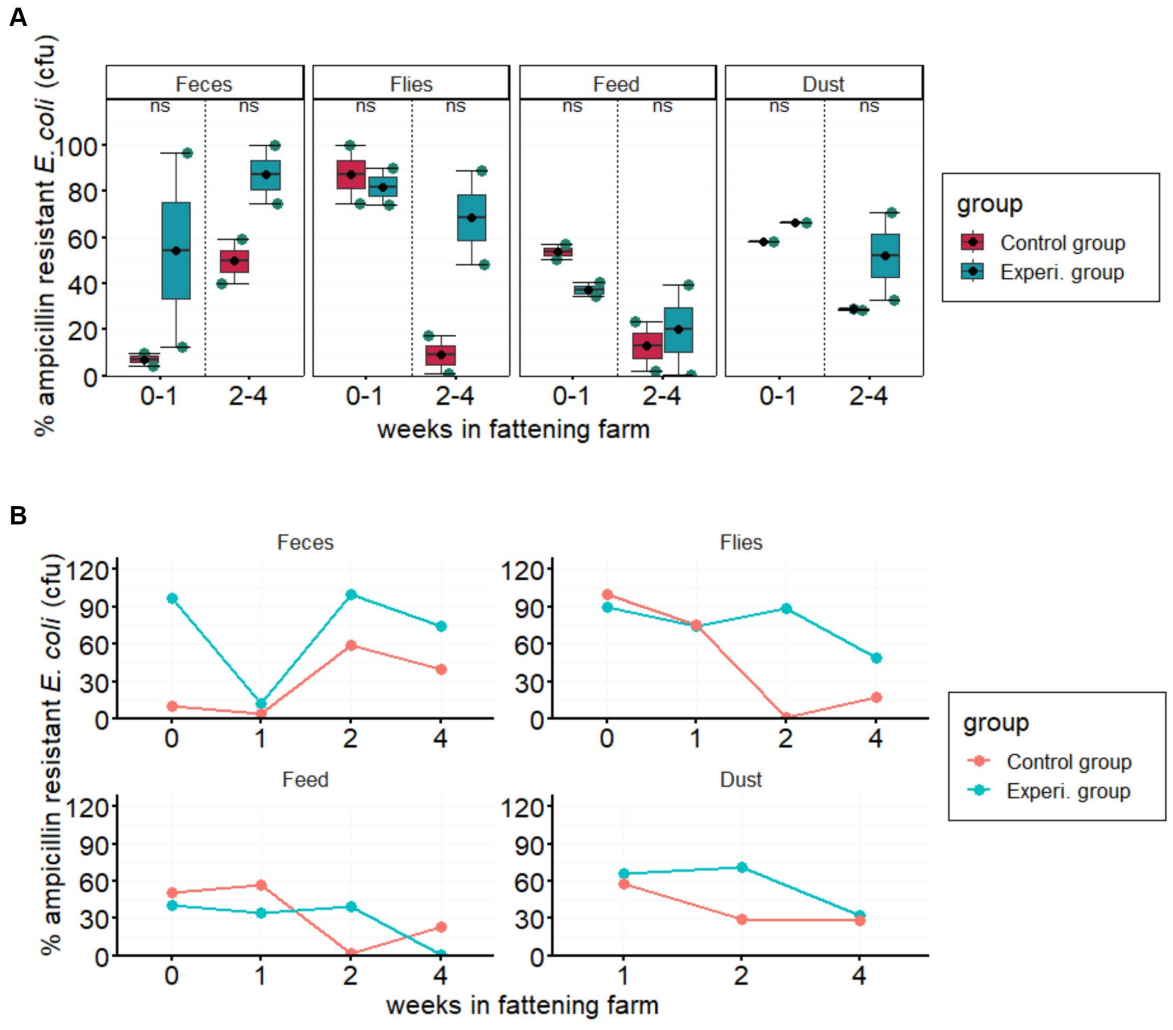
Prevalence of ampicillin resistance in the indicated sample types. **(A)** Boxplots show the % proportion of AMR *Escherichia coli* compared to total *E. coli* (in CFU/g feces, flies, feed, or dust) in the experimental and control groups. Error bars are 95% confidence intervals, the bottom and top of the box are the 25th and 75th percentiles, and the line inside the box is the 50th percentile (median). The solid blue circles in the boxplots represent the means of ampicillin-resistant *E. coli* in each group in 2-week intervals. Each column represents the according sample type. “*ns”* at the top center of each column indicates no statistically significant difference between the two groups. **(B)** Line graphs show the average weekly prevalence of ampicillin-resistant *E. coli* over the four-week study period in feces, flies, feed, and dust samples.

No statistically significant differences (*p* > 0.05) were found in the prevalence of isolated ampicillin-resistant *E. coli* between groups managed with conventional and improved hygiene (Figure 1A). However, the prevalence of resistant *E. coli* isolated from all four different sample types varied greatly throughout monitoring (<1% to 100%; Figure 1B). In particular, the proportion of AMR in the experimental groups fluctuated strongly over the study period from arrival to the fourth week, contrary to our initial hypothesis (i.e., a subsequent reduction in AMR abundance over time), which showed that factors other than hygiene management influence the spread of resistant pathogens.

**Figure 2:**
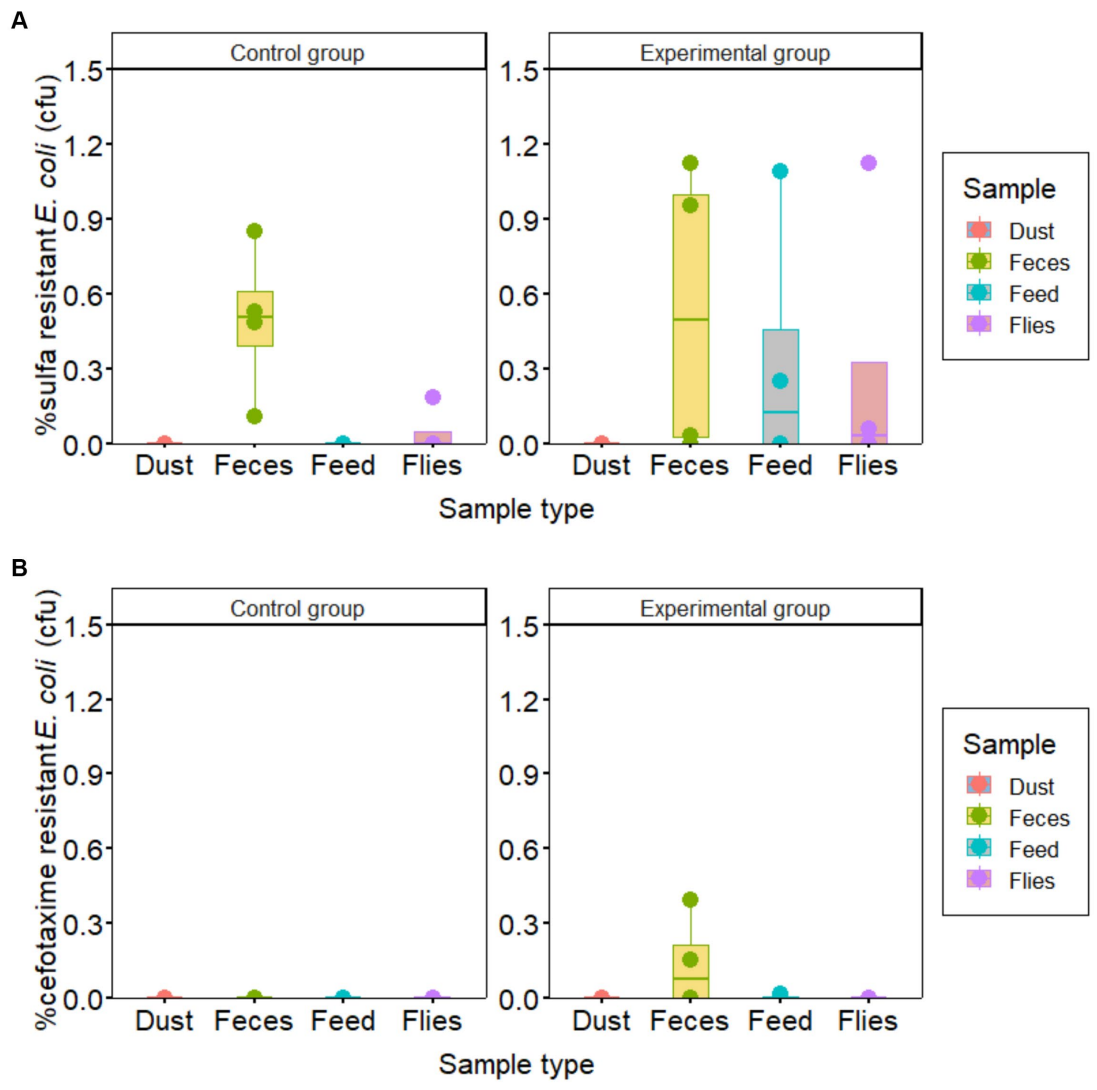
Prevalence of *Escherichia coli* resistant to **(A)** sulfonamide and **(B)** cefotaxime compared to total *E. coli* in samples collected from piglet houses for hygiene improvement assessment.

The proportions of sulfonamide-resistant *E. coli* detected in most feces, feed and fly samples were higher in the experimental groups than in the control groups. However, the proportion of sulfonamide resistant *E. coli* to total *E. coli* in both groups was less than 1.2%. Similarly, cefotaxime resistance of less than 0.5% was observed in fecal samples only in the experimental group (Figure 2).

### Antimicrobial resistance genes abundance in piglet feces

**Figure 3:**
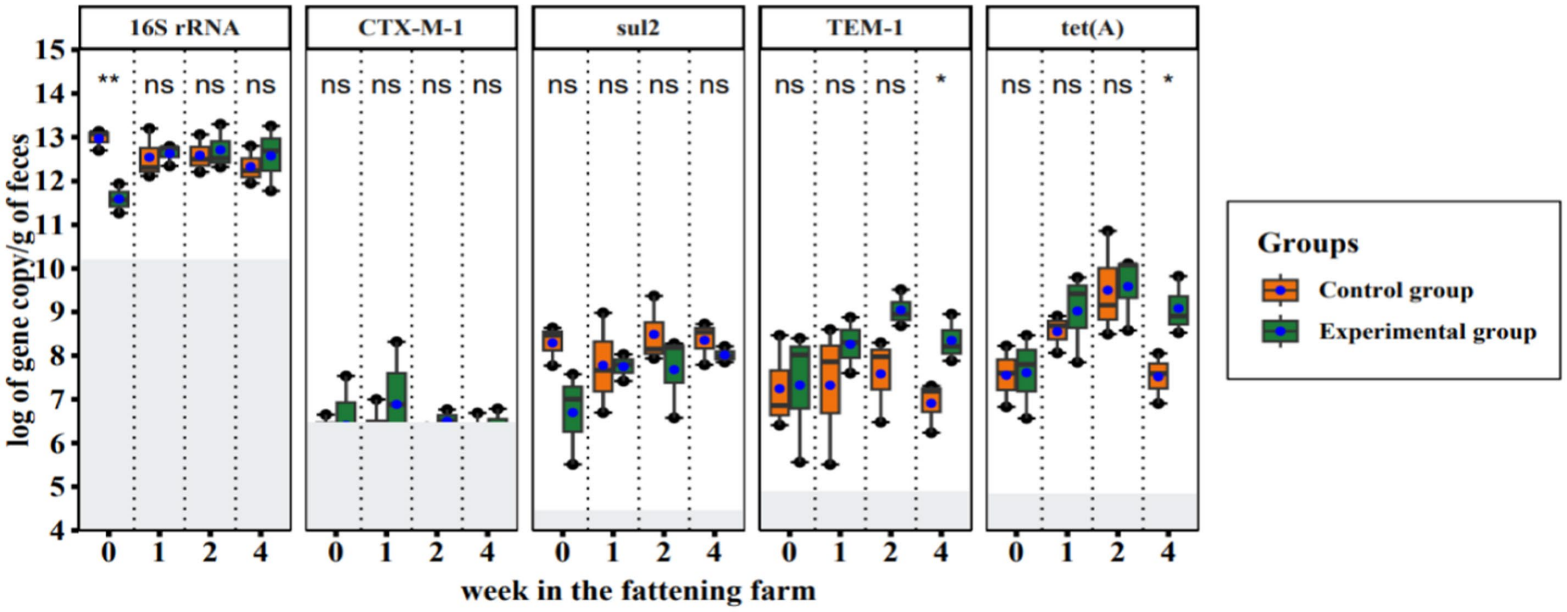
Quantitative PCR results of total bacteria and resistance genes in fecal samples collected during a hygiene improvement experiment. The X-axis represents the sampling week (week: 0, 1, 2, 4) and the Y-axis represents the logarithm of gene copies per gram of feces. Boxplots show the distribution of gene copies above the limit of quantification (LoQ); gray area = below LoQ. The bottom and top of the boxes are the first and third quartiles, respectively. The black band within the box is the median and the ends of the whisker represent the maximum (largest gene copy number) and minimum (lowest gene copy number) values. The solid blue circles indicate the average number of gene copies. Each column represents the indicated gene name and ‘ns’ indicates statistically no significant difference, while ^*^p < 0.05 and ^**^p < 0.01 show statistically significant and strong significant differences, respectively (based on Wilcoxon rank sum test).

We quantified gene copy numbers of a *16S rRNA* gene (represents the total bacteria in the fecal samples) and four ARGs [i.e., *bla_CTX-M-1_*, *bla_TEM-1_*, *sul2,* and *tet(A)*] in 24 fecal samples by qPCR in duplicates. Amplification efficiency, limit of detection, and limit of quantification were determined from the Ct values generated during each run (Supplementary Table S1). A *bla_TEM-1_*, *sul2*, and *tet(A)* were consistently high in all samples while the *bla_CTX-M-1_* gene was comparatively low and was detected in approximately half of the samples (18 out of 25). The abundance of the *16S rRNA* gene varied between the samples and covered a wide range of total bacterial gene loads ranging from 1.8 × 10^11^ to 1.9 × 10^13^ gene copies per gram of feces. We found that there were statistically significant differences in total bacteria abundance (*16S rRNA*) between all CGs and EGs in the fecal samples on the arrival date (week 0 in the fattening farm; Figure 3). However, no significant difference in total bacterial gene load was observed between both groups at the later time points. ARG abundance was not significantly different between the experimental group and the control group, except for *bla_TEM-1_* and *tet(A)* in the fourth week of the study (Figure 3).

**Figure 4:**
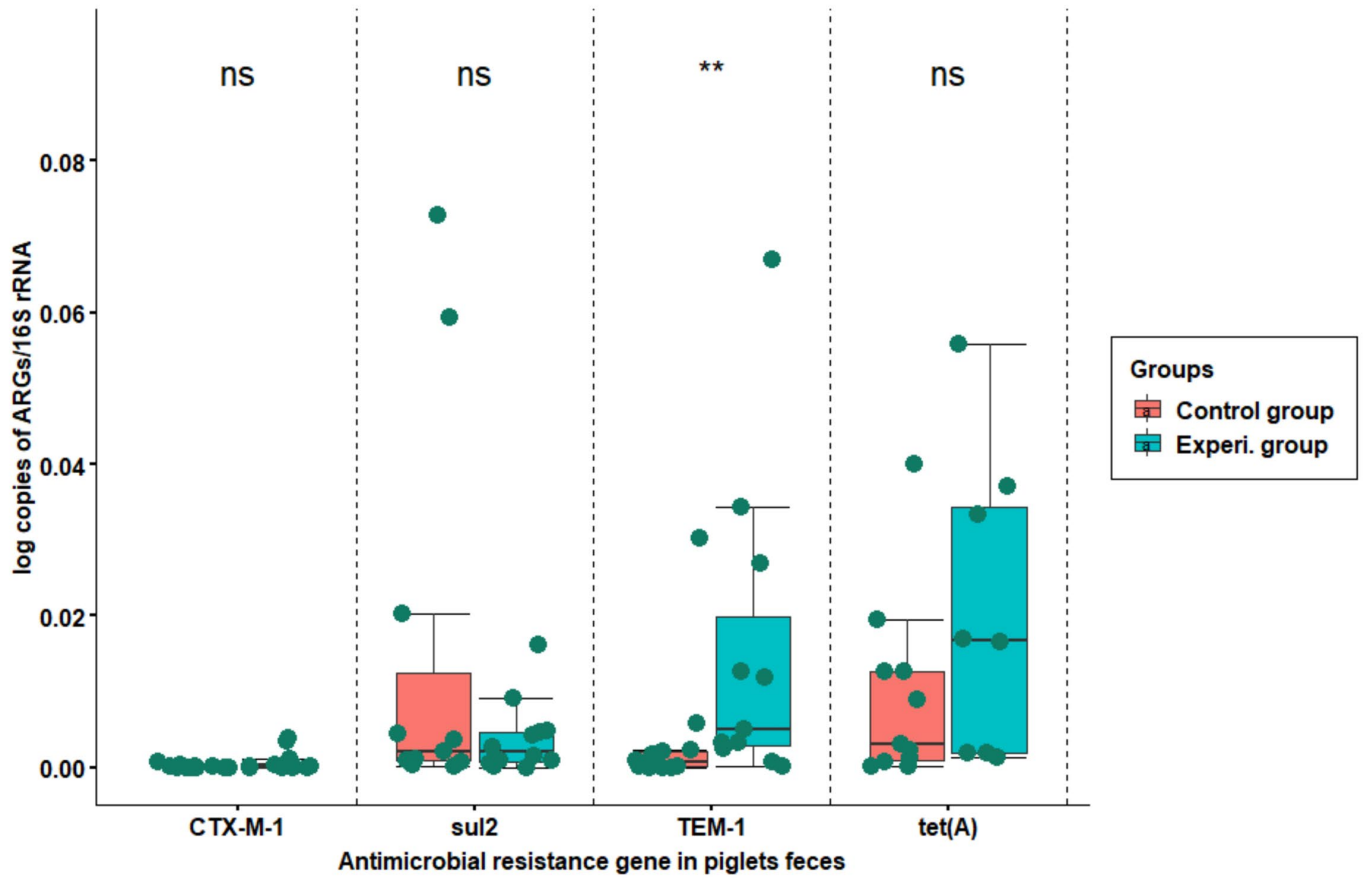
The relative abundance of resistance genes copy numbers normalized to the bacterial *16S rRNA* gene. The X-axis shows the name of resistance gene, while Y-axis represents the proportion of resistance gene copy number to *16S rRNA* gene copy number the in fecal samples. The samples were collected from flat decks where hygiene was managed using conventional techniques (red boxplot) and improved techniques (green boxplot). The boxes indicate the interquartile range of the data while the black line in the boxplot represents the median gene copy number for the samples tested. The top ‘ns’ means no significant difference, while ^**^p < 0.01 means strong significant difference (based on Wilcoxon rank-sum test).

ARG relative abundance was calculated by normalizing it to *16S rRNA* gene abundance. In the current study, the relative abundances of the *bla_CTX-M-1_* gene and *sul2* were very low compared to *bla_TEM-1_* and *tet(A).* There were no significant differences between *bla_CTX-M-1_*, *sul2* and *tet(A)* of control and experimental group. However, there was a significant difference for *bla_TEM-1_* between the control and experimental groups (Figure 4).

### Resistance genes characterization

**Figure 5:**
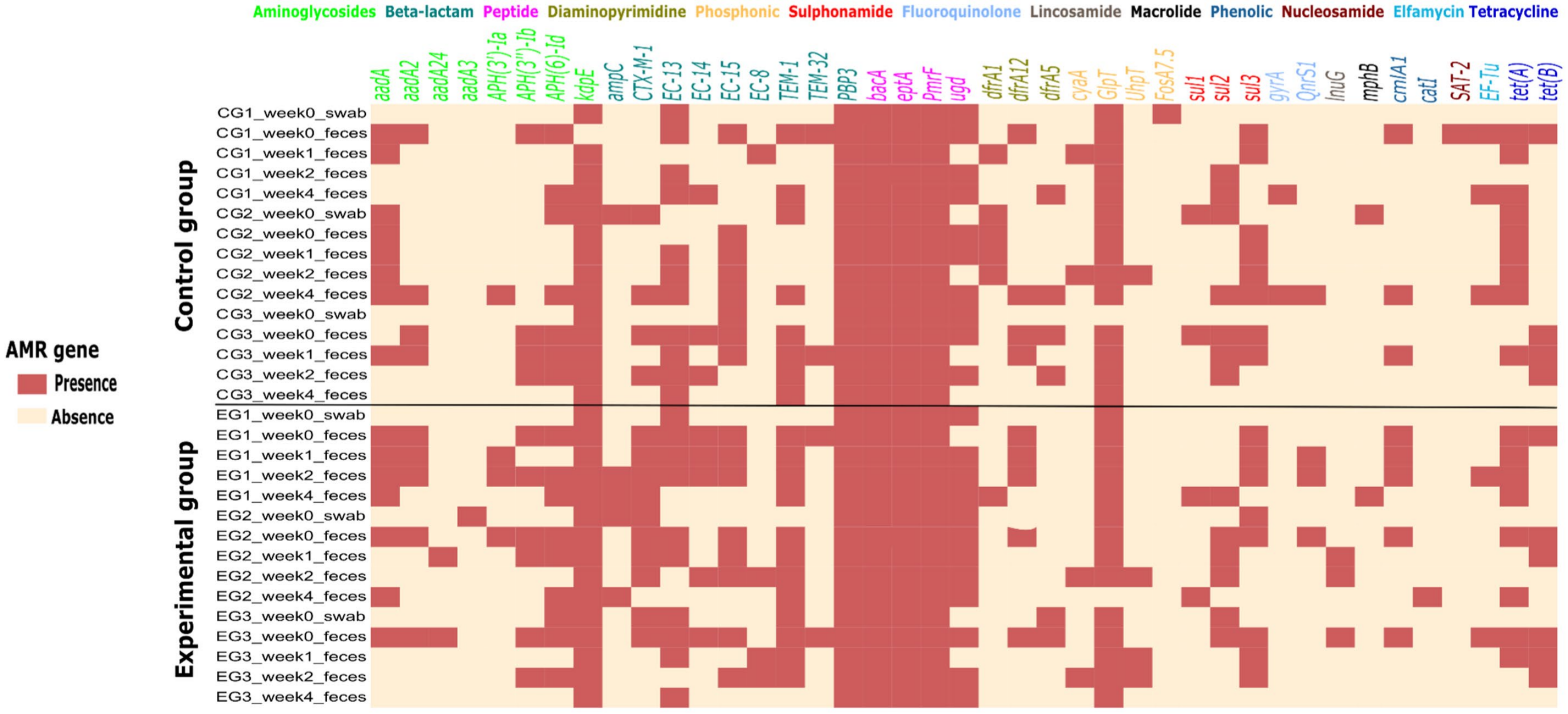
Heat map of the presence (light red) and absence (light yellow) of 41 different antimicrobial resistance genes and/or mutations in *Escherichia coli* strains isolated from 24 fecal and 6 swab samples collected weekly during the study period in experimental and control groups. At the top of the heat map are 13 classes of antibiotics to which the isolates were tested for resistance genes or mutations.

A total of 74 AMR *E. coli* isolates (68 from feces and 6 from swab samples collected for cleaning and disinfection testing) were sequenced, assembled in the Enterobase software, and antimicrobial resistance genes (ARGs) were identified using bioinformatics (analyzed using RGI) tools. About 41 ARGs and mutations against 13 different classes of antibiotics were detected in each sample with varying frequencies. All isolates were from a total of 24 fecal samples, and 6 swab samples collected from experimental and control group houses, and few isolates (1–4) from fecal samples and one from each swab. The sequenced isolates were used to develop a heat map based on ARG presence and absence, sorted for fecal and swab samples in both groups (Figure 5). The heat map shows many ARGs and mutations to aminoglycosides, beta-lactams and peptide antibiotic classes detected in the samples. Resistance associated with a target alteration of the peptide antibiotic were detected almost in all samples. Moreover, the ARGs present in the piglets’ flat decks were detected in the swab samples and after piglet arrival in fecal samples. During the four-week experimental monitoring, inconsistent ARG pattern in both groups were observed. This indirectly suggests that the majority of AMR detected in the piglet houses during the experiment may not be due to persistent AMR pathogens remaining in the house due to poor cleaning and disinfection.

### Results of disinfectant tolerance tests

**Figure 6:**
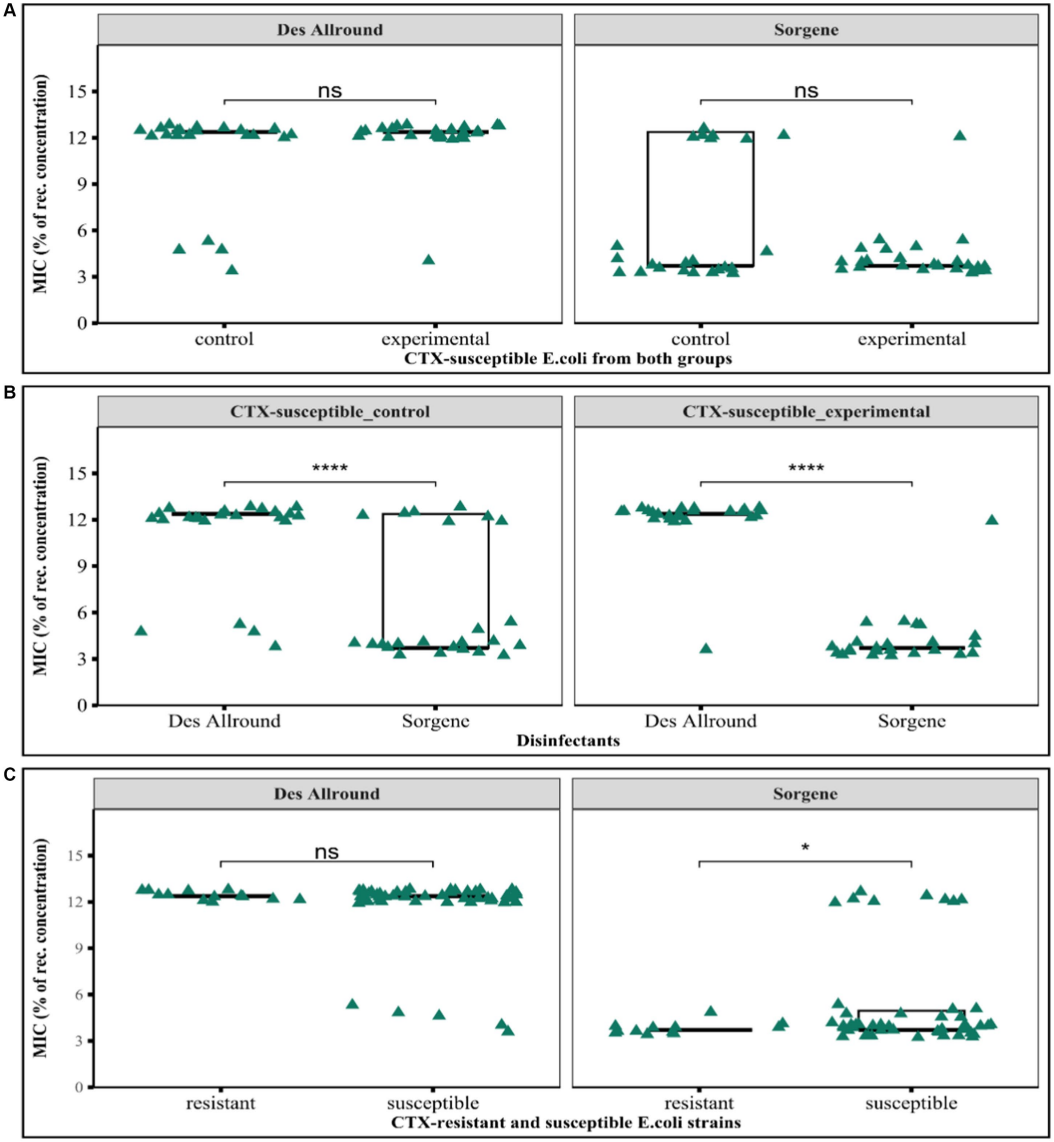
Results of the minimum inhibitory concentration determinations for the disinfectants Sorgene and Des Allround against *Escherichia coli* strains. **(A)** Comparison of susceptibility of CTX-susceptible *E. coli* isolated from control and experimental groups to both disinfectants; **(B)** Comparison of the MIC (%) of Sorgene and Des Allround against CTX-susceptible *E. coli* strains; **(C)** Comparison between CTX-susceptible and CTX-resistant to the biocidal effect of two disinfectants. ‘ns’ indicates no significant difference, while ^*^p < 0.5 and ^***^p < 0.001, means the difference is statistically significant and highly significant, respectively (based on Wilcoxon rank-sum test).

The growth inhibitory effects of the two disinfectants used in this study were determined based on measuring the minimum inhibitory concentration of the disinfectants. The two disinfectant tested here, were Sorgene (used for several years in the farm) and Des Allround (newly used in the farm during the hygiene experiments) against *E. coli* isolated in the hygiene experiment from the experimental and control group. Both CTX-susceptible and resistant *E. coli* are highly susceptible to less than 15% of the recommended concentration (Figure 6). There was no significant difference between isolates from the groups with regard to the inhibitory effect of Des Allround (*p* > 0.17) and Sorgene (*p* > 0.16; Figure 6A). We observed that higher relative concentration of Des Allround were required than Sorgene and very high significant difference in inhibiting growth of CTX – susceptible bacteria from control and experimental (*p* < 0.0001; Figure 6B). Considering median values, most bacteria were inhibited at 12.38% of the recommended concentration of Des Allround and at 3.71% of the recommended concentration of Sorgene. Cefotaxime resistant isolates were more susceptible to Sorgene than cefotaxime-susceptible strains, with maximum MIC-values of Sorgene being 4.95% in Cefotaxime-resistant and 12.38% in Cefotaxime-susceptible *E. coli* (Figure 6C).

## Discussion

In this study, we investigated the consequences of hygiene improvement on the spread of AMR *E. coli* in piglets and their environment. Poor hygiene and biosecurity in intensive livestock farming play a central role in the spread of diseases, including antimicrobial resistance, by serving as a route of transmission for pathogens (Dewulf et al., 2007; Postma et al., 2015). By improving hygiene management in the piglet house, we were unable to find a significant differences to the conventional practices in terms of reducing the presence of antibiotic-resistant bacteria.

In our mitigation trials, a high prevalence of ampicillin-resistant *E. coli* was observed in feces and fly samples from both groups (experimental and control group), while resistances were moderate in feed and relatively low in dust (Table 1). In fact, this is not surprising because enteric pathogens are primarily excreted in feces and spread via the fecal-oral route (Gerba, 2009). Flies also play an important role in the transmission of resistant pathogens through circulating pathogens from animals and/or their excretions to other individuals, feed, equipment and environment in the house, as they roam around indiscriminately and also spread outside of barns (Meerburg et al., 2007; Usui et al., 2015; Behrens et al., 2023).

Ampicillin and sulfonamide resistance were prevalent in nearly all types of samples, while very low cefotaxime (only in feces) and no ciprofloxacin resistance were detected. Ampicillin-resistant *E. coli* were present in all 24 (100%) fecal samples with a proportion between 5% and 100% to the amount of non-resistant *E. coli* (Table 1). This suggests a widespread ampicillin resistance, might be related to previous colonization (before arrival) or possibly due to the use of the drug (Dupamox; amoxicillin derivatives) against rare bacterial infections in individual piglets on the studied farm, particularly in experimental groups (Supplementary Table S1). Antibiotic treatment of individual diseased pigs resulted in increased *E. coli* resistance that can last up to some weeks (Sali et al., 2021; Tams et al., 2023). On the other hand, ampicillin is also an old antibiotic widely used in humans and animals and therefore has been heavily exposed to *Enterobacteriaceae* for many years, resulting in a high basic rate of resistance and the according resistance genes, particularly *bla_TEM-1_* (Tran-Dien et al., 2018). The *bla_TEM-1_* gene confers resistance to old beta—lactam antibiotics such as penicillin derivatives, and early cephalosporin (Salverda et al., 2010). Although no single treatment with sulfonamide drug administered to piglets in our experimental unit, sulfonamide-resistant *E. coli* were frequently detected in their fecal samples. This indicates that direct exposure to the drug is not the only factor for resistance and is more likely a consequence of sulfonamides were widely used in agriculture and livestock production over many decades (Lesch, 2007). Likewise, a previous longitudinal study indicates that the amount of antibiotics from the penicillin group (amoxicillin and ampicillin) used in weaned piglets in Germany is approximately more than 75% compared to the other antibiotics, highest treatment frequency (Schaekel et al., 2017) and also the proportion of resistance to this group of antibiotics was also the highest (Van Rennings et al., 2015; Mesa-Varona et al., 2021).

The prevalence of cephalosporin resistance in the current study was quite low (Figure 2), only 5 of 12 (42%) fecal samples from the experimental group had cefotaxime-resistant *E. coli* with a proportion less than 0.5%. Similarly, a very low frequency of *bla_CTX-M-1_* (resistance gene for extended-spectrum cephalosporins) in fecal samples was also detected by qPCR in both groups. A study by Cavaco et al. (2008) showed that the harboring of *bla*_*CTX-M*-*1*_ resistant coliform bacteria and the use of cephalosporin is the main cause of the spread of indigenous *bla_CTX-M-1_* producing *E. coli* strains and the possible emergence of strains producing *bla_CTX-M-1_* genes acquired through horizontal transfer. Therefore, the few detections of cefotaxime-resistance in the experimental groups in cultivation and qPCR could be related to exposure to *bla_CTX-M-1_*-producing bacteria before arrival or treatment with cephalosporins.

Although there was no significant difference in ampicillin resistance between improved and conventional hygiene management, some factors may have overshadowed the effect of hygiene improvement on the occurrence of AMR. As shown in Figure 1B, the proportion of ampicillin resistance observed across weeks and groups varied greatly. One such factor could be the pre-arrival colonization of piglets with ampicillin-resistant bacteria, which were detected in fecal samples on the first day of arrival (12–24 h; Supplementary Figure S2). A high proportion of ampicillin-resistant *E. coli* was observed on the arrival day, particularly in the experimental groups. In addition we observed that AMR levels can vary strongly between individuals in the same herd and of the same origin (data not shown). AMR pathogens from infected piglets can easily contaminate feed and water sources in the flat deck. The contamination of feed and water can lead to the ingestion of AMR bacteria by other piglets, thus promoting the spread of resistance easy and fast (Hutschemaekers et al., 1976). If AMR bacteria were initially detected in piglets this might have impacted hygiene management measures.

Quantitative PCR results showed that the total bacterial load in pooled fecal samples was significantly lower in the experimental group than in the control group on the first day of arrival (Figure 3). This indicates that soaking with detergents during cleaning significantly reduced the overall bacterial load in the experimental groups than just soaking with water alone in the control groups. Similarly, a former study found a significantly greater reduction in total bacteria in swab samples collected in pig houses that were first soaked with detergent compared to houses cleaned without detergent (Hancox et al., 2013). Another reason for the difference in bacterial load between the experimental and control groups can be the properties of the disinfectants. FL-des Allround (disinfectant for experimental groups) forms a foamy consistency when applied and retains the active ingredients exposed to the microbes for a longer time. On the contrary, Sorgene does not form foam and therefore drains faster and the active ingredients exposed to the microbes have comparatively less time to act. Foamy disinfectants offer a longer exposure time compared to their liquid counterparts (Cadnum et al., 2020). The time the disinfectant is exposed to the microbes is very important to the effectiveness of disinfection (Wales et al., 2021).

Selected ARGs were detected and quantified by qPCR in all fecal samples of both groups. There was no significant difference for ARGs between the samples from the experimental group and the control group, except for *bla_TEM-1_* and *tet(A)* (*p* < 0.04 for both) in the fourth week (Figure 3). In Addition, a significant difference in the relative abundance of *bla_TEM-1_* between the experimental group and the control group was detected (*p* < 0.0086; Figure 4). One possible explanation for this is that 1 week after arrival ampicillin treatment (Supplementary Table S1) given to individual piglets experimental groups 1 and 2 led to resistance selection. In the study by Zeineldin et al. (2019), authors suggested that early interventions with penicillin’s in piglets could promote resistance selection in herds. The other reason could be related to the previous colonization of the piglets with the resistant bacteria before arrival, as roughly indicated in Supplementary Figure S2.

Trends in resistance genome tracking during experiments indicate that the emergence and spread of AMR over time is not related to persistent AMR from the previous piglet batch (Figure 5). This can be determined from the ARGs in swab samples collected before the arrival of the piglets and the ARGs in fecal samples on arrival date and in the first week after arrival. The resistance gene prevalence in swab samples were very low as expected compared to those in feces. The resistance patterns in swab and fecal samples were inconsistent. We conclude that the emergence and spread of antimicrobial resistance in farms is not due to poor cleaning and disinfection. A difference between the two groups was found in the proportion of the two beta-lactam resistance genes *bla_CTX-M-1_* and *bla_TEM-1_*. This could be related to earlier colonization before arrival, and the use of antibiotics in piglets (in this study Duphamox; an amoxicillin antibiotic, was used in experimental group one) to treat bacteria results in large variations in the overall resistance frequency. Burow et al. (2019) indicated that antibiotic treatment and the spread of antibiotic resistance in the production chain have a major impact on the prevalence of antibiotic resistance in pig farms. It confirmed the difference in beta-lactam resistance of *E. coli* between treated and untreated pigs. The likelihood that piglets will carry *E. coli* resistant to ampicillin is quite high if their mothers developed resistance to the same antibiotics. Many previous studies have shown a possible link between antibiotic resistance in sows and their offspring, which could have potential effects on individual animals (Callens et al., 2014; de Greeff et al., 2020; Pholwat et al., 2020). Microorganisms originating from the maternal and surrounding environment may significantly contribute to the microbial succession observed in newborn piglets following their birth (Chen et al., 2018).

Similar to the previous argument, the distribution patterns of sulphonamide and tetracycline resistance genes could indicate that they are not group-specific, due to hygiene management or the use of both antibiotics (as shown in Figure 3; Supplementary Table S1). This shows that antibiotic exposure cannot be the only important factor in the appearance of AMR genes to these antibiotics (Holman and Chénier, 2015).

According to our hypothesis and some speculations (Ortega et al., 2013; Jean-Yves, 2018; Puangseree et al., 2021), biocide tolerance could be related to long-term use of disinfectants on farms and in some occasions linked to antibiotic resistance. However, the results of the disinfectant tolerance test showed that the *E. coli* strains isolated from both the control groups (disinfected by Sorgene) and the experimental groups (disinfected by FL-des Allround) were phenotypically susceptible to both disinfectants. Nevertheless, bacteria are able to develop resistance to disinfectants; especially when they are in the state of a spatially organized biofilm (Sanchez-Vizuete et al., 2015; Todorić et al., 2023) or are present after flushing. There was a significant difference in the minimum inhibitory concentration as a percentage of the recommended dose between the two disinfectants. To inhibit *E. coli*, a higher concentration of FL-des Allround was required than of Sorgene (Figure 6). In addition, Sorgene had a better inhibitory effect against *E. coli* that had been isolated on cefotaxime-suppelmented agar, compared to other *E. coli*, but FL-des Allround has a similar inhibitory effect on all *E. coli* isolates, irrespective of isolation. Furthermore, the bactericidal activity of Sorgene against *E. coli* isolated from the farm is quite high and no phenotypic cross-resistance with *E. coli* was observed. Similarly, Wieland et al. (2017) reported that residual concentrations of disinfectants were not able to select ESBL-/AmpC producing *E. coli*. This also agrees with Maertens et al. (2020) suggestions that repeated use of disinfectants in animal housing does not lead to antibiotic resistance or reduce susceptibility to disinfectants. Although some previous findings (Ortega et al., 2013; Schwaiger et al., 2014; Jean-Yves, 2018) indicate opportunities for bacteria to resist disinfectants, no co-(cross-)resistance between biocide and antibiotic was observed in our current study.

Barn cleaning and disinfection is of utmost importance to control the spread of antimicrobial resistance from the previous animal batch to the next one in livestock production (Martelli et al., 2017). However, the epidemiology of AMR is influenced in complex ways by a combination of factors that include antimicrobial drug use, biosecurity level, the emergence of cross-resistance, and many non-antimicrobial risk factors (Dewulf et al., 2007). Therefore, reducing the use of antibiotics and strict biosecurity measures after careful initial cleaning and disinfection have a major long-term impact on reducing antimicrobial resistance in pig farms [European Centre for Disease Prevention and Control (ECDC) et al., 2017; Mencía-Ares et al., 2021].

Compared to our previous work (Behrens et al., 2023) on the same farm, no fluoroquinolone-resistant CFUs were observed in the current study, although a proportion of fluoroquinolone-resistant CFUs of up to 10% was observed in some cases in the study before 3 years. This could be because the farm stopped using preventative antibiotics after the piglets arrived. This shows that management practices have a major influence on the emergence of antibiotic resistance. Furthermore, although all piglets in control group 2 (Supplementary Table S1) were treated with enrofloxacin during the third week of the experiment, phenotypic resistance to ciprofloxacin was not observed, even though *qnrS1* (fluoroquinolone resistance gene) was detected in the fourth week.

## Conclusion

This research contributes to understanding how hygiene and other factors influence AMR dynamics in the piglet barn. The lack of clear reduction of AMR bacteria in the improved hygiene groups compared to the control groups and the lack of resistance to the regular used disinfectants suggest that the hygiene level in the studied conventional farm was already high. The effects of an improved hygiene might be better visible in farms with a lower level of hygiene. In conclusion, while managing hygiene is essential, it alone is insufficient to significantly reduce AMR in piglet rearing. Our findings highlight the multifaceted nature of AMR spread in piglet barns and the need for a comprehensive management strategy that addresses the various contributing factors to effectively combat AMR.

## Data availability statement

The datasets presented in this study can be found in online repositories. The names of the repository/repositories and accession number(s) can be found in the article/Supplementary material.

## Author contributions

MJ: Conceptualization, Data curation, Formal analysis, Investigation, Methodology, Validation, Visualization, Writing – original draft, Writing – review & editing. VJ: Data curation, Investigation, Methodology, Validation, Writing – review & editing, Resources. BK: Formal analysis, Software, Visualization, Writing – review & editing. MB: Data curation, Investigation, Writing – review & editing, Validation. DW: Conceptualization, Methodology, Resources, Supervision, Funding acquisition, Writing – original draft. CD: Project administration, Resources, Writing – review & editing. JS: Data curation, Formal analysis, Investigation, Visualization, Writing – review & editing, Resources. CH: Conceptualization, Methodology, Resources, Supervision, Writing – review & editing, Visualization. JZ: Supervision, Writing – review & editing. TA: Conceptualization, Funding acquisition, Methodology, Project administration, Supervision, Writing – review & editing, Resources. UN: Conceptualization, Funding acquisition, Methodology, Project administration, Resources, Software, Supervision, Writing – review & editing, Validation. TK: Funding acquisition, Investigation, Methodology, Project administration, Resources, Supervision, Validation, Writing – original draft, Writing – review & editing, Conceptualization.

## Footnotes

^1^ https://enterobase.warwick.ac.uk
