# Supplementary material for: Improvements of weaned pigs barn hygiene to reduce the spread of antimicrobial resistance
Source: Front Microbiol. 2024 May 14;15:1393923. doi: 10.3389/fmicb.2024.1393923 (PMC11135127; doi:10.3389/fmicb.2024.1393923)
Supplement: Supplementary file 1 [file Table_1.docx]

**Improvements of weaned pigs barn hygiene to reduce the spread of antimicrobial resistance**

**Megarsa Jaleta^1, 6^, Vera Junker^2^, Baban Kolte^2,11^, Maria Börger^3^, Claudia Dolsdorf^4^, Julia Schwenker^5^, Christina Hölzel^5^, Jürgen Zentek^7^, Thomas Amon^1, 8^, Ulrich Nübel^2, 9, 10^ & Tina Kabelitz^1^**

^1 Leibniz Institute for Agricultural Engineering and Bioeconomy (ATB), Potsdam, Germany^

^2 Leibniz-Institute DSMZ – German Collection of Microorganisms and Cell Cultures, Braunschweig, Germany^

^3 Leibniz Centre for Agricultural Landscape Research (ZALF), Müncheberg, Germany^

^4 Teaching and Research Station for Animal Breeding and Husbandry (LVAT), Ruhlsdorf, Germany^

^5 Faculty of Agricultural and Nutritional Sciences Christian-Albrechts-University of Kiel, Kiel, Germany^

^6 Dahlem research school, Free University Berlin, Berlin, Germany^

^7 Institute of Animal Nutrition, Free University Berlin, Berlin, Germany^

^8 Institute for Animal Hygiene and Environmental Health (ITU), Free University Berlin, Berlin, Germany^

^9 German Center for Infection Research (DZIF), Partner Site Braunschweig-Hannover, Braunschweig, Germany^

^10 Braunschweig Integrated Center of SystemsBiology (BRICS), Technical University, Braunschweig, Germany^

^11 Technical University Braunschweig, Institute of Microbiology, Braunschweig, Germany^

**Supplemental information**

1. **Farm information**

The Teaching and Research Station for Animal Breeding and Husbandry (LVAT) in Ruhlsdorf, Germany rears around 800 mainly conventional pigs per year. For this study, approximately 20 - 40 piglets at the age of 21-28 days obtained from the same breeder every three weeks were housed in pre-cleaned flat decks and kept for six weeks until they were transferred to the fattening barns. The piglets were transported from the breeding farm to the LVAT using their own transport vehicle. The drivers wore company-specific clothing and shoes and had access to the barn during unloading/loading the piglets. Visitors always wear farm-specific clothing and/or shoes. The farm took hygiene breaks of at least one week to allow sufficient drying and temperature adjustment and to reduce microorganisms before the new piglets are stabled into the flat deck. There are disinfectant foot baths at the entrances to the farm.

The piglets were accommodated within a barn comprised of eight distinct flat decks. Each of these decks was further subdivided into variable numbers of small compartments or pens (Figure S1), which served to segregate groups of piglets. The flooring of these flat decks consists of slatted surfaces, constructed from mainly plastic (Figure S1). Standard cleaning and disinfection procedures are exclusively carried out after the piglets have been moved out, a process occurring at intervals of approximately 6 to 8 weeks.

Depending on their age, the piglets received specific feed types. Piglets aged between 28 and 50 days supplied with “Primastart” feed with a floury consistency. Subsequently, from 50 to 80 days “Vital” feed is provided in pellet form, while piglets exceeding 80 days get while piglets exceeding 80 days in age are allocated “Prüffutter” feed, also presented in pellet format. Across all age groups, a consistent supplementation of zinc, manganese, and copper is incorporated into their diets.

Flies are reduced by sticky flytraps. In summer and when there are too many flies, Golden (active ingredient: azamethiphos) is also used.


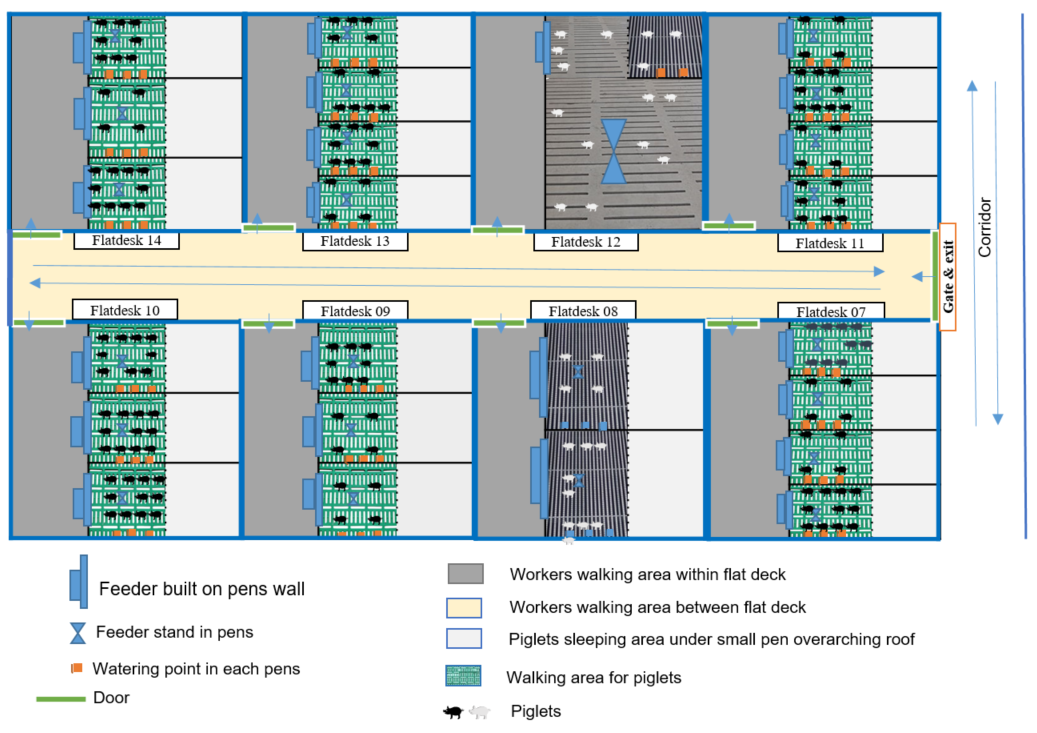


**Figure S1**. Schema of the internal flat deck compartments within the LVAT weaner pig barn.

1. **Records of the antibiotic usage during experimental hygiene activities in the farm**

In the case of isolated symptoms of illness, especially piglets with diarrhea shortly after arrival, individual treatment takes place. Such treatment often involves treatment with Borgal (a combination of sulfonamide and trimethoprim, given via single injections over a period of 3 to 5 days. Baytril (enrofloxacin) subcutaneous injection of 4 mg/kg*day for 3 days is used for bacterial infections such as meningitis. Duphamox (ampicillin derivative) is used to treat inflammation in pigs, including diseases of the claws, ears, tail and other parts of the body at a dose of 7 mg/kg body weight daily for up to 5 days. When the piglets are in the LVAT for about two to three weeks, they get vaccination against Porcine Reproductive and Respiratory Syndrome (PRRS), a respiratory disease caused by a virus and Post-Weaning Multisystemic Wasting Syndrome (PMWS) with Circoflex, a porcine circovirus type 2 (PCV2) vaccine. Deworming is done with alphamectin (active ingredient: Ivermectin).

**Table S1**: Antibiotics used to treat diseased piglets during the study period.

| **Group** | **Antibiotic** | **Disease** | **Number of days treated** | **Number treated piglets** |
| --- | --- | --- | --- | --- |
| Experimental group 1 | Duphamox (amoxicillin) | Bacterial infections | 3 days | one piglet at week-1 |
| Experimental group 2 | Duphamox (amoxicillin) | Bacterial infections | 3 days | one piglet at week-2 |
| Experimental group 3 | - | - | - | - |
| Control group 1 | - | - | - | - |
| Control group 2 | Enrofloxacin | Salmonellosis outbreak | 3 days | whole herd at week-3 |
| Control group 3 | - | - | - | - |

1. **Prevalence of ampicillin resistance on the day of arrival (12 – 24 h) at the fattening farm**

In our study, replicates exhibit high variation of antimicrobial resistance levels on the arrival date (Figure S2).


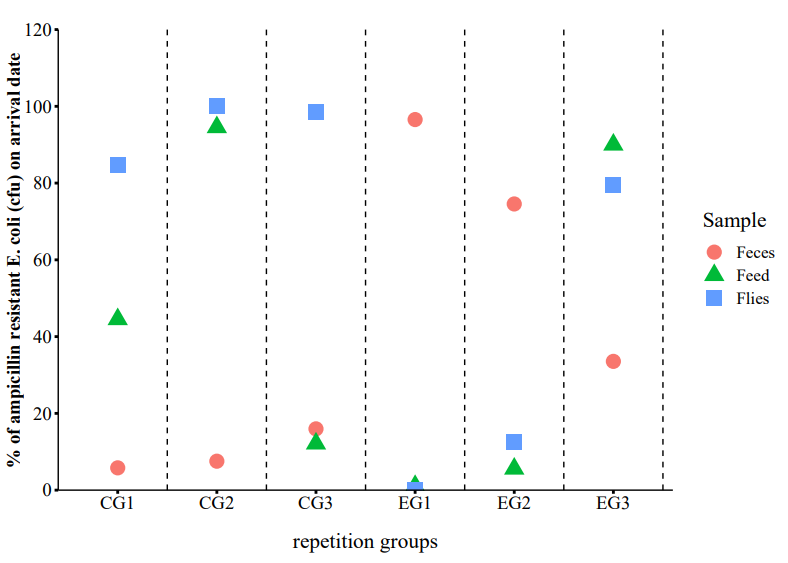


**Figure S2**: The inter-replica variation of ampicillin resistant *E. coli* in piglets on arrival, sampling within 12 to 24 hours. Note: CG = control group; EG = experimental group

1. **ARGs quantification (qPCR) parameter**

The qPCR parameters as primer pair amplification efficiency, limit of detection and limit of quantification were determined and are shown in Table S2.

**Table S2**: Primer pair efficiency (in %), determination coefficient (R^2^), detection range, LoQ, and LoD for qPCR assays.

| **Gene** | **Efficiency** | **R^2^** | **Max. & min. gene copy number in g of feces** | **LoD g^-1^ feces** | **LoQ g^-1^ feces** |
| --- | --- | --- | --- | --- | --- |
| *bla_TEM-1_* | 80±8 | 0.9995 | 3.6 x 10^5^ – 3.2 x 10^9^ | 8,28E+03 | 7,70E+04 |
| *bla_CTX-M-1_* | 90±6 | 0.9912 | 3.2 x 10^6^ – 2.0 x 10^8^ | 9,04E+05 | 3,04E+06 |
| *sulII* | 90±12 | 0.9908 | 3.2 x 10^5^ – 2.3 x 10^9^ | 5,64E+03 | 2,78E+04 |
| *tet(A)* | 80±5 | 0.9980 | 2.0 x10^6^ – 7.2 x 10^10^ | 4,39E+04 | 6,75E+04 |
| *16S rRNA* | 60±8 | 0.9945 | 1.8 x 10^11^ – 1.9 x 10^13^ | 1,29E+05 | 1,62E+10 |

1. **Supplemental data information**

**Suppl_Table1**: The proportion of *E. coli* colony forming units (% age) grown on antibiotic-containing plates.

**Suppl_Table2**: Data on antimicrobial resistance gene copies (ARGs) and their logarithmic copy number detected in fecal samples.

**Suppl_Table3**: Sequenced data of 74 *E. coli* strains (from 24 fecal and 6 swab samples) later sorted into their respective group and specific sample per week as presence (1) and absence (0) information.

**Suppl_Table4:** Results of the disinfectant susceptibility test (MIC %) for CTX-sensitive and CTX-resistant *E. coli* isolates against two disinfectants: Sorgene®Xtra and DESINTEC FL- des Allround.

**Suppl_Table5:** E**-**test result for 22 *E. coli* isolates derived from antibiotic contain MC3 agar plates based on EUCAST breakpoints
